# CDK10 functions as a tumor suppressor gene and regulates survivability of biliary tract cancer cells

**DOI:** 10.3892/or.2011.1617

**Published:** 2011-12-30

**Authors:** JIAN-HUA YU, XIANG-YU ZHONG, WEI-GUANG ZHANG, ZHI-DONG WANG, QIN DONG, SHENG TAI, HUI LI, YUN-FU CUI

**Affiliations:** 1 Department of Hepatopancreatobiliary Surgery, Second Affiliated Hospital of Harbin Medical University, Harbin 150086; 2 Department of Biochemistry and Molecular Biology, Basic Medical Science College, Harbin Medical University, Harbin 150081, P.R. China

**Keywords:** CDK10, biliary tract cancers, chemotherapy, c-RAF, cholangiocarcinoma

## Abstract

Cyclin-dependent kinase 10 (CDK10) is a member of the Cdc2 family of kinases, and has been demonstrated to be an important determinant of resistance to endocrine therapy for breast cancer. To investigate the expression and possible function of CDK10 in biliary tract cancer (BTC), we systematically examined CDK10 in tissues and cell lines. We found that expression of CDK10 was downregulated in both biliary tract tumors and cell lines. Remarkably, the expression of CDK10 correlated with clinical characteristics. Overexpression or knockdown of CDK10, respectively, inhibited or promoted cell proliferation, colony formation and migration. This suggests that CDK10 functions as a tumor suppressor gene in BTC. Overexpression of CDK10 caused malignant cells to become sensitive to chemotherapy and other hostile environments, suggesting that CDK10 functions to regulate survivability of BTC cells. We investigated the expression of six genes to resolve the mechanism. c-RAF was negatively regulated by CDK10 in both cells and specimens. Our results indicate that CDK10 plays a crucial role in the growth and survivability of biliary tract cancer, and offers a potential therapeutic target for this fatal disease.

## Introduction

Biliary tract cancer (BTC) arises from the ductal epithelium of the biliary tree, and is the second most common primary hepatobiliary malignancy (1), with a rising incidence and a dismal prognosis (1–3). This fatal disease has traditionally been divided into cholangiocarcinoma (CCA) and gallbladder cancer (GBC), which have similar pathogenesis and clinical characteristics (1). Furthermore, CCA can be classified into intrahepatic cholangiocarcinoma (ICC) and extrahepatic cholangiocarcinoma (ECC) according to the site of the tumor (1,4). ECC is divided into perihilar cholangiocarcinoma (PCC) and distal extrahepatic cholangiocarcinoma (DECC) (1). The most effective treatment for BTC is surgical resection (5); however, the disease is still fatal because patients are always diagnosed at advanced stages (6). Except surgery, both chemotherapy and radiation are used as adjuvant therapy, but the effect is still far from satisfactory (1,5). Finding effective biomarkers for earlier diagnosis, and clarifying the molecular mechanisms associated with pathogenesis and chemotherapy resistance are required to improve prognosis (5,7).

Cyclin-dependent kinase 10 (CDK10) is a member of the Cdc2 family of kinases and plays a role in the cell cycle (8). Similar to other CDKs, CDK10 contains tyrosine and threonine sites in the ATP binding domain and the phosphorylation status of these sites is crucial for determining its activity (9). Although the cyclin partner of CDK10 has not been identified, CDK10 associations have been described that play an important role in its function in the cell (9,10). CDK10 has been reported as the regulator of the Ets2 transcription factor and modulates its transactivation activity (9). In addition, the CDK10/Ets2/c-RAF signaling has been demonstrated as an important determinant of resistance to endocrine therapy for breast cancer (10). Recent studies have shown that CDK10 is a potential tumor suppressor not only in breast cancer, but also in other tumors, such as seminoma (11).

The Raf/MEK/MAPK cascade is a crucial signaling pathway for the development of CCA (12). This signaling pathway is regulated by CDK10 in breast cancer (10). In CCA and GBC, deletion or loss of heterozygosity (LOH) has been frequently detected for several regions of the long arm of chromosome 16 (13,14), where CDK10 is located (15).

In this study, we proposed that CDK10 may be a candidate tumor suppressor gene for BTC, including CCA and GBC. To support our proposals, we systematically examined the expression of CDK10 in human tumor tissue and cell lines. The impact of CDK10 expression on BTC cell biology and survivability was also evaluated by either overexpression or RNAi methods to confirm our hypothesis.

## Materials and methods

### Cell culture

HCCC-9180, SSP25 and RBE cholangiocarcinoma cell lines and the GBC-SD gallbladder cancer line were obtained from the Chinese Academy of Sciences Shanghai Branch Cell Bank (Shanghai, China). HCCC-9180, SSP25 and RBE cell lines were cultured in RPMI-1640 medium with 10% fetal bovine serum (FBS), 100 IU/ml penicillin and 100 μg/ml streptomycin. GBC-SD cells were maintained in RPMI-1640 medium with 20% FBS and antibiotics. Human intrahepatic biliary epithelial cells (BECs) and epithelial cell medium were purchased from ScienCell Research Laboratories (San Diego, CA, USA). BECs were cultured in complete medium containing 10% FBS and antibiotics. In this study, BECs were employed as the control cells for normal biliary epithelial cells.

### Plasmids, siRNA and transfection

To increase the expression of CDK10 in cell lines, pCMV6-Entry-CDK10 vector was purchased from OriGene (Rockville, MD, USA), and the ORF (open reading frame) of CDK10 was inserted into the vector. At 80–90% confluence, cells were transfected with pCMV6-Entry-CDK10 or empty vectors using Lipofectamine 2000 (Invitrogen, Carlsbad, CA, USA). To obtain stable transfectants, the cells were transfected in accordance with the aforementioned criteria. Forty-eight hours post-transfection, the cells were switched to the medium containing G418 (600 μg/ml), and the medium containing G418 was replaced every 3–4 days. After 2 weeks, isolated colonies began to appear. In 3 weeks, we selected stable transfectants expressing CDK10 for further study. The control clones expressing empty vector (Mock) were isolated at the same time.

Three siRNAs targeting CDK10 were obtained from RiboBio (Guangzhou, China) and sequences were 5′-CUGC ACAGGAACUUCAUUA-3′ (si-1), 5′-GCUCCUAUUUCA AGGAGAA-3′ (si-2), 5′-CCAGCCUCCUGGAGAAUAU-3′ (si-3), respectively. The control siRNA was also obtained from RiboBio. Twenty-four hours prior to transfection, cells were plated onto a 35-mm dish at 50–60% confluence. Transfection was performed with Lipofectamine 2000 according to the manufacturer’s protocol. The transfected cells were resuspended and cultured in regular culture medium for 48–72 h before analysis.

### Clinical tissue samples

Tissue samples were obtained from 65 patients at the Second Affiliated Hospital of Harbin Medical University from January 2007 to March 2011. Informed consent was obtained from patients and the tissue acquisition protocol was approved by the Harbin Medical University Institutional Review Board. There were 47 samples of tumor tissues (including ICC, PCC, DECC, GBC and metastasis), and 18 samples from normal bile ducts or gallbladder. Normal specimens were obtained from patients undergoing pancreatoduodenectomy because of pancreatic or duodenal diseases and whose bile ducts were disease-free. Tumor samples were obtained from CCA or GBC patients undergoing cancer-related surgery. The clinical characteristics of the patients were collected, including tumor location, histological type, differentiation grade, lymph node invasion, TNM staging and 1-year survival. Fresh tissue was frozen in liquid nitrogen and used for RNA and protein extraction.

### RNA extraction and quantitative real-time PCR

Total RNA was isolated from tissue samples or cells using TRIzol (Invitrogen Life Technologies) and total RNA was reverse transcribed to cDNA, using the PrimeScript Reagent kit (Takara, Tokyo, Japan) according to the manufacturer’s instructions. Quantitative real-time PCR was performed with a SYBR-Green kit (Takara) using the ABI 7500 Real-time PCR system (Applied Biosystems, Foster City, CA, USA). Specific primers were designed for CDK10, c-RAF, TP53 (p53), BID, ABCC2, ABCB1 and ABCB11 (Table I). Human β-actin was used as an endogenous control. The PCR procedures were performed according to the manufacturer’s instructions. All assays were performed at least three times. The relative expression levels were then determined by using the 2^−ΔΔCt^ method (16).

### Western blot analysis

Total proteins were extracted from tissue samples or cells using lysis buffer containing phenylmethyl sulfonylfluoride. Samples mixed with loading buffer were denatured, separated by electrophoresis on 12% SDS-PAGE, and then transferred to polyvinylidene fluoride membranes. The membranes were blocked with 5% non-fat milk for 2 h, and were exposed to the appropriate primary antibodies (anti-CDK10, Abgent, San Diego, CA, USA; anti-c-RAF-pS338 Phospho, Abgent; anti-PCNA, Santa Cruz Biotechnology, Santa Cruz, CA, USA; anti-β-actin, Santa Cruz Biotechnology) at an appropriate dilution for 12 h at 4°C. After three extensive times washes using TBST for 10 min each, the membranes were incubated with appropriate horseradish peroxidase-conjugated secondary antibodies (Santa Cruz Biotechnology) at a dilution of 1:8,000 for 2 h at 25°C. Immunoreactive bands were visualized with chemiluminescence. Human β-actin was employed as an endogenous control.

### Colony formation assay and wound healing assays

Twenty-four hours post-transfection with RNA oligonucleotide or plasmid DNA, HCCC-9180 and GBC-SD cells were seeded for colony formation in 35-mm dishes at a density of 200 viable cells per well. After 21 days, the cells grown in plates were fixed in 4% paraformaldehyde for 15 min. After washing, the cells were stained with 0.005% crystal violet solution for 1 h (17). The plates were aspirated, washed and allowed to air dry. Colonies were counted only if a single clone contained >50 cells. Each assay was performed in triplicate.

For wound healing assays, cells were seeded and grown to confluence on 35-mm cell culture dishes. A wound was introduced by scratching the confluent monolayer with a pipette tip (200 μl). After washing twice with PBS, serum-free medium (inhibiting cell proliferation) was added. Imaging was conducted using light microscopy at ×40 magnification, and wound healing was quantified as the average linear speed of the wound edges after 24-h incubation.

### Cell proliferation assays, chemotherapy sensitivity assays, serum-dependent growth assays and assays of tolerance to low oxygen conditions

BTC cells were transfected with RNA oligonucleotide or plasmid DNA. Five hours after transfection, equal numbers of viable cells were seeded in 96-well plates for cell proliferation assays. Cell growth was determined using the 3-[4,5-dimethylthiazol-2-yl]-2, 5-diphenyltetrazolium bromide assay (MTT) (Sigma, St. Louis, MO, USA). One-tenth volume of MTT with serum-free medium was added to each well at different time points, and the plates were further incubated at 37°C for 4 h. Formazan crystals were dissolved in DMSO. The A_590_ was measured with an enzyme-labeling instrument (BioTek, Winooski, VT, USA).

For the chemotherapy sensitivity assays, 5-fluorouracil (5-FU), epidoxorubicin (EADM), cisplatin (CDDP) and hydroxycamptothecin (HCPT) were supplied by Pharmacia Qilu (Jinan, China) and were used as the chemotherapy drugs to evaluate the effect of CDK10 on chemotherapy sensitivity. Cells that were transfected with RNA oligonucleotide (48 h post-transfection) and stable transfectants were seeded into a 96-well plate (4×10^3^ cells/well), and allowed to attach overnight. Cells were treated with various concentrations of anticancer drugs in at least six replicate wells and incubated for 48 h. The MTT assay was performed in accordance with the aforementioned criteria and dose-response curves were used to describe the results.

For studies of serum-dependence growth assays, cells were seeded onto a 96-well plate (4×10^3^ cells/well), and allowed to attach overnight. Cells were washed twice with serum-free medium, new medium with 20, 15, 10, 5 or 1% serum was added, and cells were cultured for 3 days. The MTT assay was performed in accordance with the aforementioned criteria.

In this study, we imitated low oxygen conditions using cobalt chloride to evaluate the cell survival (18). After cells were seeded and allowed to attach to a 96-well plate, the medium with cobalt chloride (final concentration of 100 μmol/l) was added. When each time point arrived, an MTT assay was performed in accordance with the afore-mentioned criteria.

### Flow cytometry for cell cycle analysis

Stable transfectants were seeded into 60-mm dishes. After 24 h of culture, to allow cells to attach, cells were treated with 5-FU, at 200 mg/l for 48 h. Cells were harvested. After washing with PBS, the cells were fixed with 80% ice-cold ethanol at 4°C overnight. After fixation, the cells were stained with 4% propidium iodide (PI) and 10% RNase A in PBS for 30 min at 37°C. A total of 2×10^4^ events were analyzed per assay by FACScan analysis using the CellQuest software (Becton-Dickinson, Franklin, NJ, USA).

### Statistical analysis

Data are presented as means ± SD from at least three separate experiments. Statistical analysis was performed by the Student’s t-test at a significance level of P<0.05. The χ^2^ test was used to show differences in categorical variables. All statistical analyses were conducted using SPSS version 11.0.

## Results

### Expression of CDK10 is downregulated in biliary tract cancer

**Figure 1:**
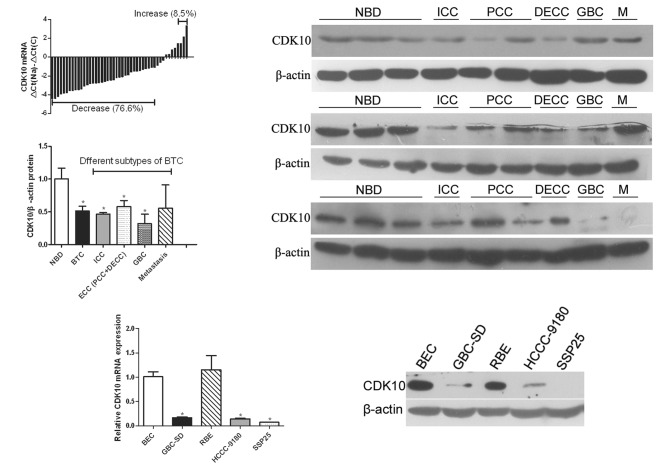
Expression of CDK10 is downregulated in BTC. (A) Real-time PCR of the expression levels of CDK10 in BTC and normal samples. ΔCt(Na), Ct value of β-actin was subtracted from that of CDK10 of every normal tissue, and the average value of the differences of all normal samples was defined as ΔCt(Na). ΔCt(C), Ct value of β-actin was subtracted from that of CDK10 of tumor. Bar value represents CDK10 mRNA level of tumor samples. Bar values <-1 indicate that the expression of CDK10 is decreased in tumors. Bar values >1 indicate that the expression of CDK10 is increased in tumors. (B) Determination of relative CDK10 protein level in 27 different samples (including 9 normal bile duct, 3 ICC, 6 PCC, 3 DECC, 3 GBC and 3 metastasis). Representative data are shown. (C) Expression level of CDK10 protein in different subtypes of BTC and normal bile duct. Student’s t-test was used to analyze the difference between the two groups. ^*^P<0.05. (D) Real-time PCR of the expression level of CDK10 in tumor cell lines. ^*^P<0.05 compared with BEC. (E) Western blot analysis of CDK10 expression in BEC and tumor cell lines. BTC, biliary tract cancer; ICC, intrahepatic cholangiocarcinoma; NBD, normal bile duct; GBC, gallbladder cancer; PCC, perihilar cholangiocarcinoma; DECC, distal extrahepatic cholangiocarcinoma; M, metastases; BEC, biliary epithelial cells. β-actin was employed as an internal control for western blot analysis or real-time PCR.

To determine the clinical relevance of CDK10 in human BTC, quantitative real-time PCR was performed to determine the expression of CDK10 mRNA in human cancer and normal tissue samples. The average expression of 18 normal samples was defined as the basic expression of normal tissues. Forty-seven tumor samples (including ICC, PCC, DECC, GBC and metastasis) were examined and showed that decreased CDK10 mRNA occurred in 36 of the 47 samples (76.6%), compared with normal tissue (Fig. 1A). The mRNA levels of CDK10 were significantly different between tumors and normal tissues (P=0.00075). We also determined the protein levels of CDK10 by western blotting in 27 clinical samples (including normal tissues, ICC, PCC, DECC, GBC and metastasis). As shown in Fig. 1B, decreased CDK10 protein occurred in 15 of 18 (83.3%) tumors, compared with normal tissues (Fig. 1B). Statistical analysis indicated that CDK10 protein was significantly different between tumors and normal tissues (P=0.004). In subtypes of BTC, ICC and GBC had a significantly downregulated CDK10 expression, compared with normal tissues (P=0.01 and 0.048, respectively; Fig. 1C). In PCC and DECC specimens, the expression of CDK10 had a tendency to decrease, but was not marked (P=0.123 and 0.119, respectively). However, the difference between ECC (including PCC and DECC) and normal specimens was marked (P=0.04, Fig. 1C).

Patients were divided into three groups according to the expression of CDK10 at the mRNA level. The clinical characteristics were compared in these groups. Lower expression of CDK10 was significantly associated with worse TNM staging, increased lymph node invasion, and higher serum carbohydrate antigen (CA)19-9 level in BTC (Table II; P<0.05), but not with age, gender, histology type, tumor location, differentiation grade, serum carcinoembryonic antigen (CEA) level, hepatitis B virus (HBV) infection, metastasis, and 1-year survival (Table II).

We also compared the expression of CDK10 between four different tumor cell lines (GBC-SD, HCCC-9180, SSP25 and RBE) and normal BECs. Real-time PCR analysis revealed that three cell lines (GBC-SD, HCCC-9180 and SSP25) expressed lower levels of CDK10 compared to BECs; the difference was significant (Fig. 1D). Western blot analysis was also performed and revealed a similar result to that of real-time PCR analysis (Fig. 1E).

### Overexpression of CDK10 inhibits proliferation of biliary tract cancer cells

**Figure 2:**
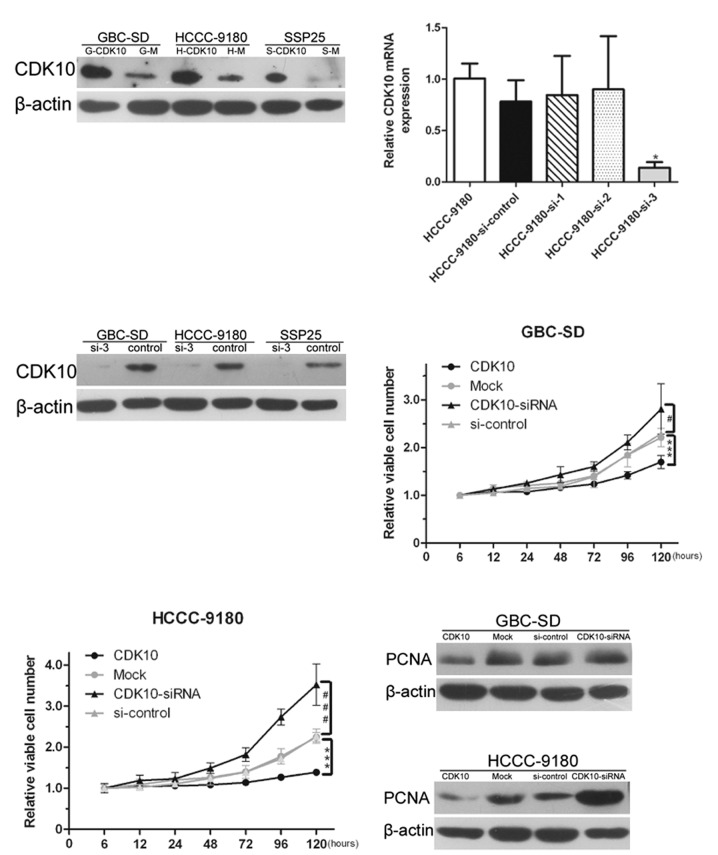
Overexpression of CDK10 inhibits BTC cell proliferation. (A) Western blot analysis of CDK10 expression levels in stable transfectants (G-CDK10, H-CDK10 and S-CDK10) and mock (G-M, H-M and S-M) of three different cell lines (GBC-SD, HCCC-9180 and SSP25). (B) Real-time PCR analysis of CDK10 mRNA level in HCCC-9180 transfected with different siRNA or control RNA. ^*^P<0.05 vs. the cells transfected with control RNA. (C) Three different cells lines were transfected with siRNA (si-3) or control RNA, respectively. Seventy-two hours after transfection, western blot analysis was performed confirm the effect of transfection. (D and E) The proliferation of GBC-SD and HCCC-9180 were examined by MTT assays at different time points after transfection with RNA oligonucleotide (CDK10-siRNA or si-control) or plasmid DNA (CDK10 or Mock). The data are presented as mean ± SD. ^***^P<0.001 vs. mock cells. ^#^P<0.05; ^###^P<0.001 vs. cells transfected with si-control RNA. (F and G) The protein expression levels of PCNA in GBC-SD and HCCC-9180 were examined by western blot analysis at 72 h after transfection. BTC, biliary tract cancer. β-actin was employed as an internal control for western blot analysis or real-time PCR.

Three cell lines (GBC-SD, HCCC-9180 and SSP25) that expressed lower levels of CDK10 were transfected with pCMV6-Entry-CDK10 or empty vectors. After 3 weeks, stable transfectants were obtained and named as H-CDK10 (HCCC-9180), G-CDK10 (GBC-SD) and S-CDK10 (SSP25). The control clones expressing empty vector (Mock) were named as H-M (HCCC-9180), G-M (GBC-SD) and S-M (SSP25), respectively. Western blotting was performed to confirm positive clones (Fig. 2A).

Three siRNAs targeting CDK10 were designed and transfected into cells. Real-time PCR was performed to confirm which one was the most effective. As shown in Fig. 2B, si-3 was used for further experiments because this siRNA was most effective in suppressing CDK10 expression. Seventy-two hours after transfection with si-3, total protein was extracted from cells, and western blotting was performed to confirm the effect of RNAi (Fig. 2C).

Given that CDK10 is downregulated in clinical specimens and that it may act as a tumor suppressor, we decided to examine whether CDK10 had anti-oncogenic functions in BTC cells *in vitro*. To determine whether CDK10 regulated tumor growth, we performed cell proliferation assays with GBC-SD and HCCC-9180 cells. MTT assays were performed at the indicated time points after transfection. Overexpression of CDK10 significantly inhibited the proliferation of GBC-SD and HCCC-9180 cells (P<0.001; Fig. 2D and E). Conversely, silencing of CDK10 clearly promoted the proliferation of HCCC-9180 and GBC-SD cells (P<0.001 and 0.05, respectively; Fig. 2D and E). Total proteins were extracted from stable cell lines and the cells that were transfected with siRNA after 72 h. Western blot analysis was performed and proliferating cell nuclear antigen (PCNA) was employed as a reporter of proliferation. The results demonstrate that the lack of CDK10 increased proliferation. Conversely, the proliferation was inhibited in CDK10-overexpressing cells (Fig. 2F and G).

### Silencing of CDK10 promotes colony formation and migration of cells

**Figure 3:**
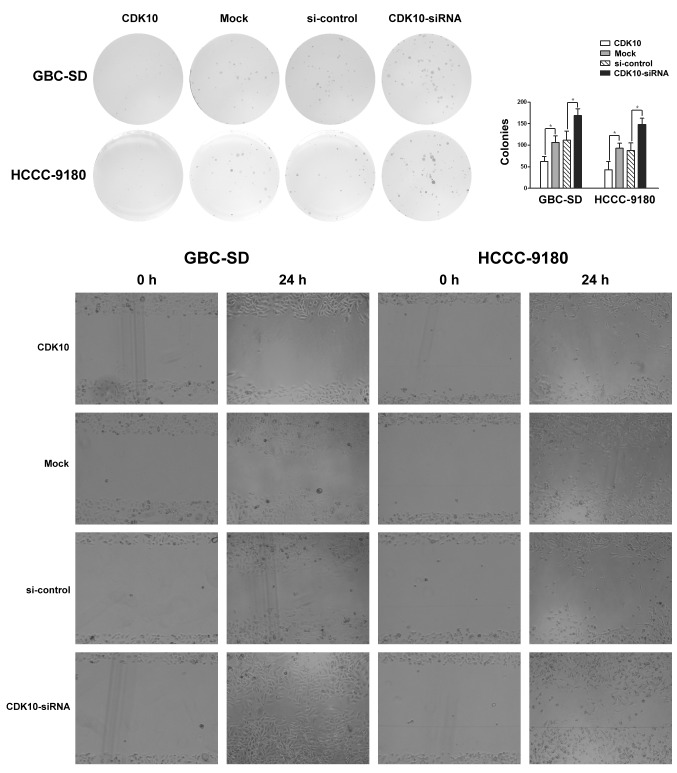
CDK10 inhibits colony formation and migration of BTC cells. (A and B) Overexpression or knockdown of CDK10, respectively, inhibited or promoted colony formation. Colonies were counted only if a single clone contained >50 cells in the 21 days after seeding. ^*^P<0.05. (C) Wound healing assay was used to identify the role of CDK10 in migration. Both GBC-SD and HCCC-9180 were used in this analysis. BTC, biliary tract cancer.

As shown in Fig. 3A, overexpression or knockdown of CDK10, respectively, attenuated or promoted colony formation of GBC-SD and HCCC-9180 cells (Fig. 3A and B; P<0.05). Greater numbers of colonies of larger size were formed by knockdown of CDK10 as compared with control RNA, whereas CDK10-overexpressing cells formed only a few small colonies (Fig. 3A and B).

We next investigated the function of CDK10 in the migration of GBC-SD and HCCC-9180 cells. CDK10 knockdown resulted in a significant increase in cell migration, compared to cells transfected with control RNA (Fig. 3C). As expected, CDK10 overexpression induced a significant decrease in cell migration (Fig. 3C). These observations suggest that CDK10 inhibited the migration of tumor cells.

### Overexpression of CDK10 reverses the resistance to chemotherapy for biliary tract cancer and decreases survivability of biliary tract cancer cells

**Figure 4:**
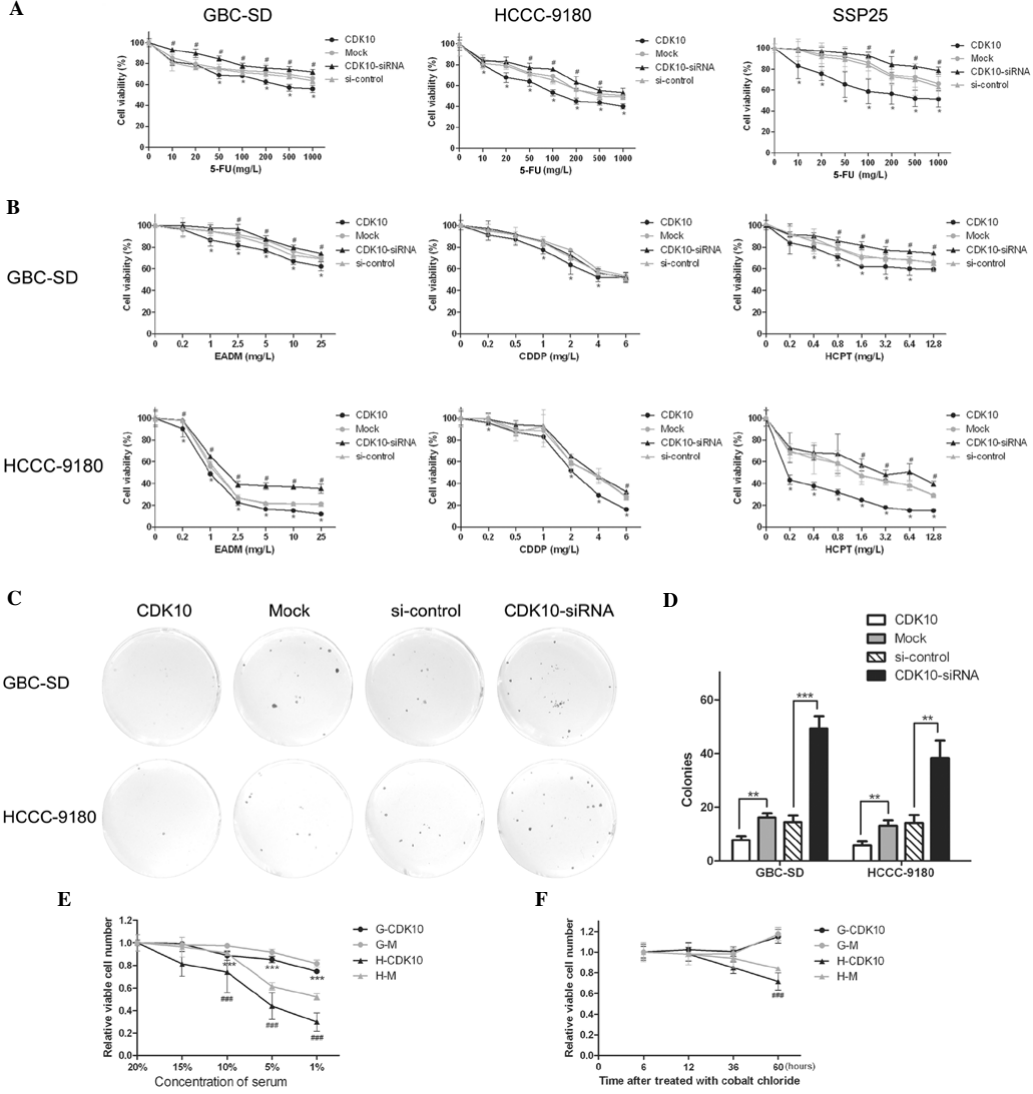
Overexpression of CDK10 decreases the survivability of BTC cells to chemotherapy, serum starving and hypoxia. (A and B) The cell viability assay was performed in both CDK10-overexpressing and CDK10-silenced cells in the 48 h after treated with chemotherapeutic drugs. GBC-SD, HCCC-9180 and SSP25 were treated with 5-FU. GBC-SD, HCCC-9180 were treated with EADM, CDDP and HCPT. All the data are presented as mean ± SD of four determinations/experiment from three separate experiments. ^*^P<0.05 compared with mock cells; ^#^P<0.05 vs. the cells transfected with control RNA. (C and D) Forty-eight hours after exposed to 5-FU (200 mg/l), the colony forming assay was performed to confirm the result of cell viability assay. Colonies were counted in accordance with aforementioned criteria. ^**^P<0.01; ^***^P<0.001. (E and F) Serum-dependence growth assays and hypoxia-tolerant assays were performed in CDK10-overexpressed cells (G-CDK10 and H-CDK10) and mock cells. (G-M and H-M). MTT assays were used to examine viable cells. Data shown are the mean ± SD of three independent experiments. ^***^P<0.001 vs. G-M cells; ^###^P<0.001 vs. H-M cells. BTC, biliary tract cancer; 5-FU, 5-fluorouracil; EADM, epidoxorubicin; CDDP, cisplatin; HCPT, hydroxycamptothecin.

Given that CDK10 has been identified as an important determinant of resistance to endocrine therapy for breast cancer (10), we decided to examine whether CDK10 influenced resistance of BTC cells to chemotherapy. 5-FU was used as the major drug and the GBC-SD, HCCC-9180 and SSP25 cell lines were examined. As shown in the dose-response curves (Fig. 4A), CDK10 silencing significantly decreased sensitivity to 5-FU for all three cell lines, and overexpression of CDK10 increased sensitivity to 5-FU, conversely. To confirm whether the influence of CDK10 was specific to 5-FU or common to other chemotherapeutic drugs, EADM, CDDP and HCPT were used as additional drugs, and GBC-SD and HCCC-9180 cell lines were tested. For both GBC-SD and HCCC-9180 cells, after overexpression or silencing of CDK10, EADM, CDDP and HCPT showed similar alteration as 5-FU (Fig. 4B). The result indicated that expression of CDK10 influenced resistance to chemotherapy rather than specifically to 5-FU.

To obtain more evidence, we performed colony-forming assays with chemotherapy in 24-well plates (100 cells/well). After 24-h culture, to allow cells to attach, cells were treated with 5-FU, at 200 mg/l for 48 h. Cells were fed with fresh medium without 5-FU after washing with PBS and the other procedures were the same as for the ordinary colony-forming assay. Overexpression of CDK10 induced potentiation of the inhibitory effect of 5-FU on the colony-forming ability. Furthermore, the inhibitory effect of 5-FU was relieved because of silencing of CDK10 (Fig. 4C and D).

We investigated the survivability of CDK10-overexpressed cells by serum-dependence growth assays and hypoxia-tolerant assays. The serum-dependence growth assays showed that CDK10-overexpressed cells were much more independent of serum (Fig. 4E). In hypoxia-tolerant assays, CDK10-overexpressed HCCC-9180 cells showed a significantly smaller decrease in survival, compared with Mock group. However, GBC-SD was not sensitive to cobalt chloride (Fig. 4F). Taken together, these results suggest that CDK10 regulates the survivability of BTC cells.

### An increase in CDK10 expression induces G_0_/G_1_ cell cycle arrest and potentiates the cell cycle arrest induced by 5-FU

**Figure 5:**
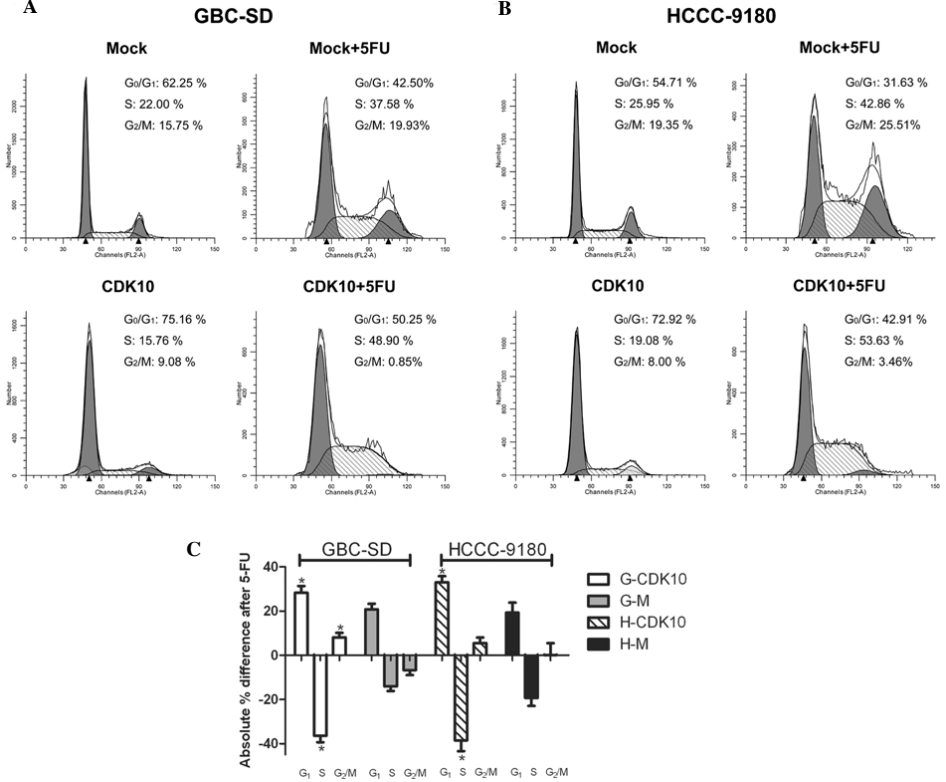
Resistance to 5-FU in CDK10-overexpressed cells is characterized by increased 5-FU-induced G_1_ arrest. (A and B) Stable transfectants of both GBC-SD (A) and HCCC-9180 (B) were used in the analysis. Cell cycle profiles were assessed by propidium iodide (PI) staining and fluorescence-activated cell scanning (FACScan) from cell aliquots taken both before and after treatment with 5-FU. (C) Absolute differences were calculated by subtracting the percentage of cells in each phase of the cell cycle before 5-FU treatment from the percentage of cells in each phase after 5-FU treatment. Significant differences in G_1_ and S phases after 5-FU treatment were observed in two kinds of CDK10-overexpressing cells, indicating increased 5-FU-induced G_1_ arrest. Data shown are the mean ± SD of three independent experiments. Data shown are the mean ± SD. ^*^P<0.05 vs. mock cells (G-M and H-M, respectively).

In colon cancer cells, the antiproliferative effect of 5-FU results in induction of cell cycle arrest at the G_1_ phase, and is characterized by an increase in the number of cells in the S phase (19). To investigate the possibility that an increase in CDK10 expression enhances the 5-FU-induced cell cycle arrest, cell cycle profiles were assessed. Compared with Mock cells, CDK10-overexpressed cells showed a significant increase in the number of G_1_ phase cells and a simultaneous significant decrease in G_2_/M phase cells (Fig. 5A and B). Mock cells (G-M and H-M) treated with 5-FU displayed the expected increase in S phase populations (Fig. 5A and B). In contrast, 5-FU-treated, CDK10-overexpressing cells (G-CDK10 and H-CDK10) showed a significant increase in the number of G_1_ phase cells and a significant decrease in S phase cells compared with Mock cells (Fig. 5). More interesting, G-M cells showed an increase in the number of G_2_/M phase cells after treatment with 5-FU, while the number of G-CDK10 cells in G_2_/M phase showed a significant decrease (Fig. 5C).

### CDK10 is a negative regulator of expression of c-RAF in biliary tract cancer

**Figure 6:**
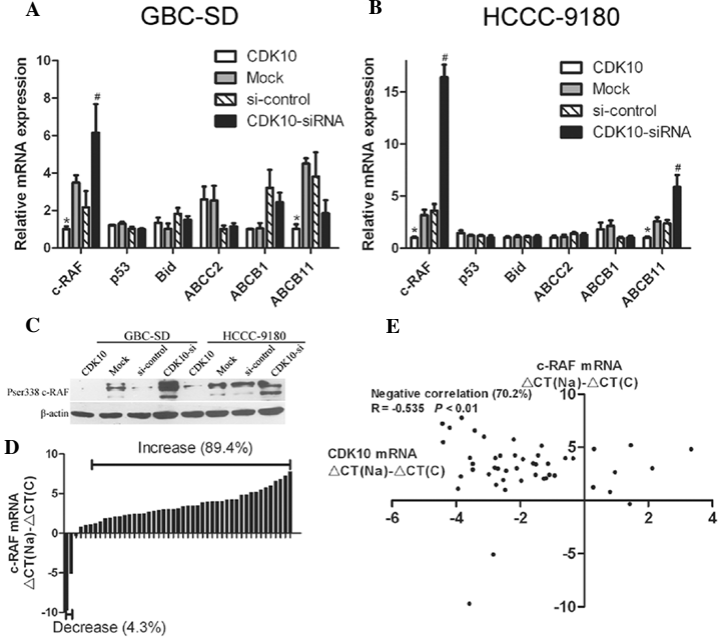
CDK10 is a negative regulator of expression of c-RAF in BTC. (A and B) Real-time PCR was performed to examine the expression level of six genes which were supposed to be regulated by CDK10. ^*^P<0.05 vs. mock cells; ^#^P<0.05 vs. cells transfected with si-control RNA. (C) Western blot analysis of CDK10 expression levels in CDK10-overexpressed and CDK10-silenced cells. (D) Real-time PCR showed the expression level of c-RAF in BTC samples and normal samples. The data are presented as ΔCt(Na)-ΔCt(C) in accordance with the defined in Fig. 1A. (E) Increased c-RAF is correlated with decreased CDK10 in BTC. Both CDK10 and c-RAF mRNA expression levels of all clinical samples were shown in the panel. Inversely correlated samples were counted only if CDK10 value was <-1 and c-RAF value was >1 at the same time (second quadrant). Correlation coefficient (R) between c-RAF and CDK10 mRNA levels was calculated in inversely correlated samples. BTC, biliary tract cancer. β-actin was employed as an internal control for western blotting or real-time PCR.

To investigate the mechanism for the alteration of resistance to chemotherapy, we examined five genes involved in mechanisms of resistance (20) including TP53 (p53), BID, ABCC2, ABCB1 and ABCB11. Given that c-RAF has been identified as a target protein regulated by CDK10 and influences resistance to endocrine therapy for breast cancer, c-RAF was also examined (10). The result of quantitative real-time PCR showed that CDK10-overexpressing cells (G-CDK10 and H-CDK10) had decreased c-RAF mRNA levels, compared with Mock cells (P<0.01; Fig. 6A and B). In contrast, CDK10-silenced cells showed increased c-RAF mRNA, compared with cells transfected with control RNA (P<0.01; Fig. 6A and B). Among the other five candidate genes, only ABCB11 showed an expected alteration of expression in HCCC-9180 cells, but not in GBC-SD cells (Fig. 6A and B). The protein levels of c-RAF were examined in transfected cells by western blotting. The results of western blotting corresponded to those of real-time PCR (Fig. 6C), and demonstrated that c-RAF was regulated by CDK10 in both GBC-SD and HCCC-9180 cells.

We supposed that c-RAF was regulated by CDK10 in BTC in the same manner as described for breast cancer (10). Thus, we investigated the expression of c-RAF mRNA in human cancer samples and normal samples by real-time PCR. Increased c-RAF mRNA occurred in 42 of 47 (89.4%) cancer samples, compared with normal tissues (Fig. 6D). The mRNA levels of c-RAF were significantly different between tumors and normal tissues (P=0.0363). Furthermore, we also investigated the correlation between the expression level of c-RAF and clinical characteristics. As shown in Table II, higher expression of c-RAF was significantly associated with worse TNM staging, increased lymph node invasion and poorer differentiation grade, but not with age, gender, serum CA19-9 level, and 1-year survival (Table II). More interesting, an increase in c-RAF mRNA with a simultaneous decrease in CDK10 mRNA occurred in 33 of 47 (70.2%) cancer samples. Statistical analysis indicated that decreased CDK10 was correlated with increased c-RAF (Fig. 6E). These data indicate that CDK10 is a negative regulator of expression of c-RAF in BTC.

## Discussion

A previous study has reported that CDK10 expression is reduced in breast cancer and CDK10 silencing induces resistance to endocrine therapy (10). The reason why CDK10 is downregulated in breast cancer with aberrant DNA methylation is still controversial (10,21), CDK10 is being investigated as a tumor suppressor (10,11). However, little is known about the expression and function of CDK10 in BTC.

We showed that CDK10 was aberrantly expressed in BTC samples and cell lines, which demonstrates that expression of CDK10 is downregulated in BTC, and that it functions as a tumor suppressor to influence the cellular processes of BTC cells. Although biliary cancers include ICC, ECC and GBC, CDK10 was downregulated in all of them (Fig. 1C). Inhibition of CDK10 expression induced aberrant activation of growth, migration and survivability (including resistance to chemotherapy, serum starvation and hypoxia tolerance) of BTC cells. More interesting, lower expression of CDK10 and higher expression of c-RAF were significantly associated with clinical characteristics, such as worse TNM staging and more lymph node invasion. The results suggested that expression of CDK10 could be used as a candidate index to evaluate BTC.

Resistance to chemotherapy is one of the major limiting factors for the application of chemotherapeutic drugs in BTC (1,5). Based on CDK10-silencing-induced resistance to endocrine therapy (10), we investigated the correlation between CDK10 and chemotherapy. We found that increased CDK10 was correlated with decreased resistance to chemotherapy. These findings may contribute to improving the effect of chemotherapy in the future.

To explain the alteration in resistance to chemotherapy induced by CDK10, we investigated the expression of several candidate genes. The results confirmed that the expression of c-RAF is regulated by CDK10 in BTC cells similarly to breast cancer cells (10). Overexpression of c-RAF has been reported to induce MAPK pathway activation (22,23). Aberrant activation of the MAPK pathway induces aberrant growth and increases the threshold for cell death (24,25), resulting in increased survivability of tumor cells. We examined the correlation between CDK10 and c-RAF in clinical samples, which was not confirmed in previous research. The results indicated that CDK10 was a negative regulator of c-RAF in cells and clinical samples. Our results indicate that CDK10 may function in cellular processes, at least partially, through c-RAF.

Inactive CDK10 has been shown to lead to a G_2_/M arrest in mammalian cells, but wild-type CDK10 only shows a modest effect (8). However, in our study, we investigated two malignant cell lines, and showed CDK10-overexpressing cells had an increase in the G_1_ phase of the cycle, compared with the control group. Coincidentally, in MCF-7 breast cancer cells, it has been reported that there is a significant decrease in the number of G_1_ phase cells, in the absence of tamoxifen treatment, because of CDK10 silencing (10). CDK10 is not only a kinase but also a negative regulator of Ets2 transcription factor (9,10). Ets2 has been found to play a role in controlling the cell cycle though regulating Cdc2 expression (26). The expression of c-RAF is the more noteworthy factor, because it is regulated by CDK10 and plays an important role in the cell cycle (10,27). Taken together, the CDK10/Ets2/c-RAF signaling may help explain why such an unusual event occur ed in the G_1_ phase. More interestingly, after treatment with 5-FU, CDK10-overexpressing cells showed a significantly increase in the G_1_ phase cells in both the GBC-SD and HCCC-9180 cell lines, and a significant decrease in the G_2_/M phase cells in GBC-SD. We suggest that the overexpression of CDK10 induces more cells to remain at a resting stage and makes cells sensitive to chemotherapy in BTC.

In conclusion, we report that expression of CDK10 is downregulated in biliary tract cancer and that it functions as a tumor suppressor. CDK10 restoration inhibits tumor growth, cell migration and survivability, and induces malignant cells to become sensitive to chemotherapy in the biliary tract cancer. These functions are at least partially mediated via a negative regulation of c-RAF, thus offering a potential therapeutic approach for treatment of biliary tract cancer with low expression of CDK10.

## Figures and Tables

**Table I:** Primer sequences.

Symbol	Forward (5′-3′)	Reverse (5′-3′)
CDK10	TGGACAAGGAGAAGGATG	CTGCTCACAGTAACCCATC
ACTB	CAGCACAATGAAGATCAAGATC	GTGTAACGCAACTAAGTCATAG
RAF1	TCAGGAATGAGGTGGCTGTTCTG	CTCGCACCACTGGGTCACAATT
TP53	CCTCAGCATCTTATCCGAGTGG	TGGATGGTGGTACAGTCAGAGC
BID	TGGGACACTGTGAACCAGGAGT	GAGGAAGCCAAACACCAGTAGG
ABCC2	GCCAACTTGTGGCTGTGATAGG	ATCCAGGACTGCTGTGGGACAT
ABCB1	GCTGTCAAGGAAGCCAATGCCT	TGCAATGGCGATCCTCTGCTTC
ABCB11	TTACAAGAACTCCAGATTCC	TGATAAGTACTGCGACAGC

**Table II:** Relationship between CDK10 or c-RAF expression and clinicopathological features of biliary tract cancer.

		CDK10				c-RAF			
			
Variables	N	Low	Moderate	High	P-value	Low	Moderate	High	P-value
Gender									
Male	34	28	4	2	0.311	1	1	32	0.213
Female	13	8	3	2		1	2	10	
Age (years)									
≥60	17	14	2	1	0.776	2	1	14	0.158
<60	30	22	5	3		0	2	28	
Tumor location									
ICC	6	6	0	0	0.711	0	0	6	0.695
PCC	20	15	4	1		1	3	16	
DECC	12	9	1	2		1	0	11	
GBC	5	3	1	1		0	0	5	
Metastases	4	3	1	0		0	0	4	
Histology type									
Adenoma	42	34	5	3	0.122	1	3	38	0.159
Non-adenoma	5	2	2	1		1	0	4	
Lymph node invasion									
Present	26	25	1	0	**0.002**	0	0	26	**0.031**
Absent	21	11	6	4		2	3	16	
TNM staging									
I–II	15	6	6	3	**<0.001**	0	3	12	**0.023**
II–IV	32	30	1	1		2	0	30	
Serum CEA level (ng/ml)									
>5	10	10	0	0	0.144	1	0	9	0.407
≤5	37	26	7	4		1	3	33	
Serum CA19-9 level (U/ml)									
>37	31	28	3	0	**0.003**	2	1	28	0.292
≤37	16	8	4	4		0	2	14	
Differentiation									
G1	9	6	2	1	0.182	1	3	5	**0.003**
G2	19	12	4	3		0	0	19	
G3	19	18	1	0		1	0	18	
HBV infection									
+	23	17	4	2	0.890	0	0	23	0.068
−	24	19	3	2		2	3	19	
Metastasis after surgery									
+	13	11	1	1	0.712	2	0	11	0.135
−	14	10	2	2		0	2	12	
Survival (year)									
>1	16	11	2	3	0.281	0	2	14	0.116
≤1	11	10	1	0		2	0	9	

The CDK10 or c-RAF expression level of clinical samples was defined in accordance with the criteria mentioned in Fig. 1A. ICC, intrahepatic cholangiocarcinoma; PCC, perihilar cholangiocarcinoma; DECC, distal extrahepatic cholangiocarcinoma; GBC, gallbladder cancer; TNM, tumor-node-metastasis classification according to the AJCC/UICC 7th edition; CEA, carcinoembryonic antigen; CA, carbohydrate antigen; G1, well differentiated; G2, moderately-differentiated; G3, poorly-differentiated; HBV, hepatitis B virus. The χ^2^ test was used to show differences of categorical variables. P-values in bold show statistically significant differences.

## Abbreviations

CDK10: cyclin-dependent kinase 10
BTC: biliary tract cancer
CCA: cholangiocarcinoma
GBC: gallbladder cancer
ICC: intrahepatic cholangiocarcinoma
ECC: extrahepatic cholangiocarcinoma
PCC: perihilar cholangiocarcinoma
DECC: distal extrahepatic cholangiocarcinoma
MAPK: mitogen-activated protein kinase
PCNA: proliferating cell nuclear antigen
